# Prevalence and risk factors of emergence agitation among pediatric patients undergo ophthalmic and ENT Surgery: a cross-sectional study

**DOI:** 10.1186/s12887-023-04434-y

**Published:** 2023-11-24

**Authors:** Hong Yu, Xiaohui Sun, Ping Li, Xiaoqian Deng

**Affiliations:** 1 Department of Anesthesiology, West China Hospital, Sichuan University, No.37 Guoxue Alley, Chengdu, 610041 China; 2 Department of Anesthesiology, West China School of Nursing, West China Hospital, Sichuan University, Chengdu, 610041 China; 3 Department of Anesthesiology, Wu’an First People’s Hospital, Handan, 056300 China

**Keywords:** Emergence agitation, General anesthesia, Pediatrics, Risk factor, Ophthalmic Surgery, Otorhinolaryngology Surgery

## Abstract

**Background:**

Some studies reported that pediatric patients undergoing otorhinolaryngology (ENT) and ophthalmic surgeries have higher incidences of emergence agitation (EA). Children with EA tend to carry the risk of self-harm, have longer periods of recovery and delayed hospital discharge. Consequently, EA needs to be monitored and risk factors ought to be emphasized to implement preventative measures. The objective of this study was to describe EA and to identify risk factors after pediatric ophthalmic or ENT surgery.

**Methods:**

Between September 2021 and December 2021, a cross-sectional study was conducted in 100 children aged of 0–12 years who underwent ophthalmic or ENT surgery. The Watcha scale was used to observe and record EA, which was defined at levels of 3 or 4 at any time in the post-anesthesia care unit (PACU). The pain intensity was graded with the Face, Legs, Activity, Cry, Consolability (FLACC) Scale after surgery. Patient and surgery-related characteristics, the behavioral criteria of EA, the pharmacologic and non-pharmacologic interventions and recovery outcomes were objectively recorded. A binary logistic regression model was constructed to identify the associated factors of EA.

**Results:**

From the 100 analyzed children, 58 were males and 42 were females, and 44 patients received ophthalmic surgery and 56 ENT surgery. The median age was 6 (IQR 4–7) years. The overall incidence of EA among pediatrics was 30% (34.5% for ENT and 24.4% for ophthalmic surgery). High preoperative modified Yale Preoperative Anxiety scale (m-YPAS) grade (OR = 1.19, 95%CI 1.06–1.33, P = 0.003) and high postoperative FLACC score (OR = 3.36, 95%CI 1.88–6.02, P < 0.001) were risk factors for EA.

**Conclusions:**

This study identified that preoperative anxiety and postoperative pain are associated with EA in children after ophthalmic or ENT surgery. Preoperative anxiety assessment and management, and administration of adjunct analgesic treatments should be considered in the routine care.

## Introduction

Emergence agitation (EA) is a cluster of postoperative disturbing behaviors during early recovery from general anesthesia and patients with EA may present with restlessness, crying, excitation, inconsolability, thrashing, and agitation [1]. The term emergence delirium (ED) has been used interchangeable with EA in several studies [2, 3]. However, ED is not fully equivalent to EA, as ED can present with hypoactive signs or mixed forms and hyperactive signs similar to agitation [4–6]. Patients who experience EA may suffer secondary harm due to displacement of surgical dressings, removal of intravenous access, disruption of surgical closure, and necessity for restraint by nurses [1, 7]. These patients frequently require additional interventions and may have a prolonged length of stay in PACU and even after short surgical procedures [8].

The etiology of EA is multifactorial [9]. Several factors have been suggested, including patient characteristics, preoperative anxiety, anesthetic agents used, pain and type of surgery [10–13]. EA is more common in children than in adults and the incidence of EA was reported inversely correlated with age in children [14, 15]. Besides, studies reported that pediatric patients undergoing otorhinolaryngology (ENT) and ophthalmic surgeries have higher incidences of EA [7, 16, 17]. In particular, EA in patients undergoing ENT surgery may increase the risk of airway obstruction and hypoxemia due to anatomical characteristics of operative location [13]. Beyond the risk of self-harm, longer periods of recovery and delayed hospital discharge, the prevalence of EA is perhaps a relevant indicator of quality of care. Consequently, EA needs to be monitored and risk factors ought to be emphasized to implement preventative measures, when applicable, to reduce incidence and prevent adverse consequences. The aim of this study was to determine the incidence of EA and its associated risk factors among children who underwent ophthalmology and ENT procedures at a major tertiary hospital in China.

## Methods

### Study design

This was a cross-sectional exploratory study conducted between September 2021 and December 2021 in West China Hospital of Sichuan University. The study was approved by the Ethics Committee of West China Hospital (No. 2021 − 973) and registered at chictr.org.cn (ChiCTR2100050982) on September 9, 2021.

### Participants

Eligible study subjects were pediatric patients aged 0–12 years old, American Society of Anesthesiologists (ASA) levels of I to II and admitted to the PACU after undergoing an elective ENT or ophthalmic surgery. The investigators obtained written informed consent from the legal guardian of children before the start of any protocol-specified procedures or assessments. Children with no parental consent and history of neurological and behavioral disorders were excluded.

### Procedure

No premedication was administrated to the children. At pre-anesthetic period, parents were allowed in the pre-holding area but not during induction. The modified Yale Preoperative Anxiety scale (m-YPAS) score, was measured by a trained nurse at the pre-holding area which was able to accurately assess preoperative anxiety levels in children [18]. It consists of 22 items divided into five categories, including activity, vocalizations, emotional expressivity, apparent awakening state, and family interaction. Higher rating corresponded to higher level of anxiety, and a score of > 30 indicates anxiety. All patients were routinely monitored with electrocardiogram, pulse oximetry, non-invasive blood pressure and end tidal carbon dioxide during anesthesia procedure. Anesthesia management protocol was based on the discretion of the anesthesiologist. At the end of the surgical procedure, all children were transferred to the PACU without extubation. During PACU, all routine monitoring was performed. Parents were not allowed to attend to their child in the PACU. After confirming spontaneous and smooth respiration with sufficient tidal volume and purposeful movement, the endotracheal tube was removed by the attending anesthesiologist. The pharmacologic (propofol 1 mg/kg or fentanyl 0.5-1ug/kg) and/or non-pharmacologic interventions (words to reassure, physical comfort, video distraction, playing with toy) were administered when the PACU bedside anesthesiologists deemed the children to be needed according to their clinical experience and dose of analgesic used during operation. The patients were discharged according to PACU discharge criteria with routine practice (Alderete score ≥ 9) and at the discretion of the PACU nurse.

### Data collection

The data were recorded in a case report form including demographic characteristics, m-YPAS score, surgery-related characteristics, the behavioral criteria of agitation and related data in PACU and in the ward. Surgery‐related data such as surgical type, duration of surgery and anesthesia, the medications used intraoperatively, and recovery duration in PACU were recorded.

After transfer to PACU, all emergence behaviors were observed and recorded by a trained nurse every 10 min after extubation and at the time of discharge of PACU. The Watcha scale was used to observe and record EA. The Watcha scale consists of four levels: level 1 = child is calm, level 2 = crying, but can be consoled, level 3 = crying, but cannot be consoled, or level 4 = agitated and thrashing around [19]. A level of 3 or 4 recorded at any time in PACU was considered as EA. Patients were screened for sedation using the Ramsay sedation score with scores of 1(irritability), 2(consciousness and cooperative), 3(deeper sleep and more agile response), 4(lighter sleep with faster awakening time), 5(sound sleep with slow response), and 6(no response) points, respectively [20]. The pain intensity was graded with the Face, Legs, Activity, Cry, Consolability (FLACC) Scale in PACU and 2, 4, 6, 24 h after surgery in the ward. The scale has 5 categories (face, legs, activity, cry, consolability). Each category is scored on the 0–2 scale, which results in a total score of 0–10 (0 = relaxed and comfortable; 1–3 = mild discomfort; 4–6 = moderate pain; 7–10 = sever discomfort or pain or both) [21].

Postoperative anesthetic complications, including respiratory depression (SpO_2_ below 90% or where intervention is required to help the child breathe (i.e. mandibular support, airway placement or use of an auxiliary bag-mask breathing device), nausea, vomiting, laryngospasm, bronchospasm, and other adverse symptoms were recorded. The data collectors were supervised by the principal investigator (XQD) throughout the entire data collection process.

### Statistical analysis

A sample size calculation was not done as this was an exploratory observational study. All data were entered into a Microsoft Office Excel spreadsheet and statistical analyses were conducted using the SPSS statistical software (Version 25; SPSS Inc., NY, USA). Descriptive statistics were used to characterize the participating children. The continuous variables were described in median and interquartile range (IQR). The continuous variables analyses were conducted using the Student’s t-test. The categorical variables were summarized by number and percentages of patients and analyses were conducted using Pearson’s Chisquare, Fisher’s exact test or Mann-Whitney U test for non-parametric date. Multivariate correlation analysis was performed by logistic regression analysis and the adjusted odds ratio (OR) [95% confidence interval (CI)] was presented. All p-values less than 0.05 were considered statistically significant.

## Results

### Participant’s characteristics

One hundred pediatric patients aged 0–12 years old with ASA class I-II were enrolled, among whom 58 were males and 42 were females. The median age was 6 (IQR 4–7) years, with 45% of 4–6 years old. A total of 45 patients underwent ophthalmic surgery and 55 ENT surgery. The median m-YPAS was 23.7 (23.3–26.7). Other patient characteristics are shown in Table 1.

**Table 1:** Baseline characteristics

Child characteristics	n (%)/median (IQR)
Gender	
Male	58(58%)
Female	42(42%)
Age (year)	6(4–7)
Infant (< 1 year)	1(1%)
Toddler (1–3 years)	17(17%)
Preschooler (4–6 years)	46(46%)
School (> 6 years)	36(36%)
BMI (kg/cm^2^)	15.4(14.3–16.6)
ASA physical class	
I	6(6%)
II	94(94%)
Complications	10(10%)
Preop m-YPAS grade	23.7(23.3–26.7)

ASA, American Society of Anesthesiologists; BMI, body mass index; IQR, interquartile range; m-YPAS, modified Yale Preoperative Anxiety Scale

Intraoperative medications are shown in Table 2. About opioid analgesics, 81children received fentanyl or sufentanil and 26 received hydromorphone. Glucocorticoids were given to 83 children and 82 received 5-HT_3_ receptor antagonist as antemetics. All patients received sevoflurane for anesthesia maintenance.

**Table 2:** Intraoperative variables

Variables	n (%)/median (IQR)
**Surgical type**	
Ophthalmic surgery	45(45%)
Strabismus surgery	40(40%)
Intraocular lens implantation	3(3%)
Tumor resection	2(2%)
ENT surgery	55(55%)
Adenotonsillectomy	52(52%)
Cochlear implantation	2(2%)
Thyroglossal cyst resection	1(1%)
Induction drug*	
Midazolam	91(91%)
Propofol	91(91%)
Sevoflurane	98(98%)
Maintenance drug*	
Sevoflurane	100(100%)
Propofol	1(1%)
Intraoperative analgesia*	
Only fentanyl/sufentanil	74(74%)
Only hydromorphone	19(19%)
Fentanyl/sufentanil + hydromorphone	7(7%)
Lidocaine	19(19%)
Glucocorticoids	83(83%)
5-HT_3_ receptor antagonist	82(82%)
Surgical time (min)	30(22–42)
Anesthesia time (min)	56(48–73)

ENT, otorhinolaryngology; IQR, interquartile range. *Non-mutually exclusive

### EA and its risk factors

The overall incidence of EA among pediatrics was 30% (34.5% for ENT and 24.4% for ophthalmic surgery). From 30 patients with EA, 19 (32.7%) were males and 11 (26.2%) females. The EA was detected in 9 (50%) patients in age group of 0–3 years, 14(30.4%) in 4–6 years and 7(19.4%) in > 6 years, respectively. EA was significantly associated with more propofol administration (30% vs. 0%, OR = 0.70, 95%CI 0.55–0.89, P < 0.01), more non-pharmacological intervention (15.7% vs. 56.7%, OR = 0.51, 95%CI 0.34–0.78, P < 0.01), and higher FLACC score in PACU (1.9 ± 2.5 vs. 8.2 ± 2.4, MD=-6.3, 95%CI -7.3 to -5.2, P < 0.01). Complications in PACU and in the ward, PACU stay and hospital stay were comparable between EA patients and non- EA patients (Table 3).

**Table 3:** Outcomes in PACU and in the ward between EA and non-EA patients

Variables	Overall (n = 100)	Non-EA group (n = 70)	EA group (n = 30)	P value
**Pharmacological interventions in PACU**				
Propofol	9(9%)	0(0)	9(30%)	<0.01
Fentanyl	6(6%)	2(2.9%)	4(13.3%)	0.064
**Non-pharmacological intervention in PACU**	28(28%)	11(15.7%)	17(56.7%)	<0.01
1 intervention	10(10%)	6(8.6%)	4(13.3%)	
2 interventions	13(13%)	4(5.7%)	9(30%)	
3 interventions	5(5%)	1(1.4%)	4(13.3%)	
**Maximal FLACC score in PACU**	3.8 ± 3.8	1.9 ± 2.5	8.2 ± 2.4	<0.01
0	38(38%)	37(52.9%)	1(3.3%)	<0.01
1–3	11(11%)	11(15.7%)	0(0)	
4–6	27(27%)	21(30%)	6(20%)	
> 6	24(24%)	1(1.4%)	23(76.7)	
**Maximal FLACC score in the ward**	0.7 ± 1.9	0.5 ± 1.6	1.2 ± 2.5	0.211
0	83(83%)	60(85.7%)	23(76.7%)	0.049
1–3	8(8%)	7(10%)	1(3.3%)	
4–6	7(7%)	2(2.9%)	5(16.7%)	
> 6	2(2%)	1(1.4%)	1(3.3%)	
**Complications in PACU**				
Severe sedation*	23(23%)	19(27.1%)	4(13.3%)	0.133
Nausea/vomiting	2(2%)	0(0)	2(6.7%)	0.088
Rescue analgesics	6(6%)	2(2.9%)	4(13.3%)	0.064
Respiratory depression	0(0%)	0(0%)	0(0%)	NA
Laryngospasm	0(0%)	0(0%)	0(0%)	NA
Bronchospasm	0(0%)	0(0%)	0(0%)	NA
**Complications in the ward**				
Nausea/vomiting	8(8%)	6(8.6%)	2(6.7%)	1.000
Dizziness	9(9%)	9(12.9%)	0(0)	0.093
Sore throat	1(1%)	1(1.4%)	0(0)	1.000
**Preop m-YPAS grade**	28.7 ± 11	27.2 ± 10.2	32.0 ± 12.1	0.065
**Extubation time (min)**	21.7 ± 10.3	22.2 ± 9.2	20.5 ± 12.6	0.456
**PACU stay (min)**	55.8 ± 14.5	55.1 ± 13.5	57.4 ± 16.6	0.479
**Hospital stay (day)**	1.75 ± 0.87	1.7 ± 1.0	1.7 ± 0.6	0.960

The categorical variables were summarized by number and percentages of patients. The continuous variables were described in mean ± standard deviation. EA, emergence agitation; FLACC, Face, Legs, Activity, Cry, Consolability; PACU, post-anesthesia care unit. *Defined as Ramsay sedation scale score of 5–6

The baseline and perioperative variables were entered into multivariate analysis when their univariate p value was < 0.1 or considered factors that contribute to EA. Univariate analyses showed that participants who received hydromorphone had the lower incidence of EA, compared with those who did not (11.5% vs. 36.5%, OR = 4.40, 95%CI 1.21–16.05, P = 0.025).

Multivariate analysis showed that high preoperative m-YPAS grade (OR = 1.19, 95%CI 1.06–1.33, P = 0.003) and high postoperative FLACC score (OR = 3.36, 95%CI 1.88–6.02, P < 0.001) were risk factors associated with EA (Table 4).

**Table 4:** Univariate and Multivariate analysis of variables related with EA

	Univariate analysis		Multivariate analysis	
Variables	OR (95% CI)	P-value	Adjusted OR (95% CI)	P-value
**Gender (Female)**	0.73(0.30–1.76)	0.480	0.19(0.03–1.44)	0.108
**Age**	0.81(0.66–0.99)	0.038	1.32(0.85–2.07)	0.219
**Preop m-YPAS grade**	1.04(0.99–1.08)	0.056	1.19(1.06–1.33)	0.003
**FLACC score in PACU**	2.33(1.62–3.34)	< 0.001	3.36(1.88–6.02)	< 0.001
**Intraop sufentanil/fentanyl**	0.38(0.01–1.40)	0.144	-	-
**Intraop hydromorphone**	4.40(1.21–16.05)	0.025	2.67(0.34–20.9)	0.349
**Intraop lidocaine**	1.77(0.54–5.87)	0.345	-	-
**Surgery type (eye)**	0.61(0.26–1.48)	0.275	-	-
**Surgical time**	0.99(0.97–1.02)	0.633	-	-
**Anesthesia time**	0.99(0.98–1.01)	0.664	-	-

CI, confidence interval; EA, emergence agitation; ENT, otorhinolaryngology; FLACC, Face, Legs, Activity, Cry, Consolability; m-YPAS, Modified Yale Preoperative Anxiety scale; OR, odds ratio; PACU, post-anesthesia care unit

## Discussion

Our study demonstrated that higher preoperative m-YPAS grade and postoperative FLACC score were associated with higher incidence of EA. That is, preoperative anxiety and postoperative pain were significantly correlated with the occurrence of EA in pediatric patients who underwent ENT or ophthalmic surgery. Also, EA patients received more pharmacological and non-pharmacological intervention during recovery period.

The incidence of postoperative EA varies by diagnostic tool, surgical type, age group and anesthetic drug. To date, several scales have been proposed as tools for assessing EA in children and the accurate clinical instruments to diagnose EA in children is not well known [14]. We used a 4-point Watcha scale as it was the most expedient and practical scale which was proved to be correlated reasonably well with the Pediatric Anesthesia Emergence Delirium (PAED) scale. In addition, the PAED scale has disadvantages of inherent subjectivity in assessing each behavior item [22] and controversial cutoff point for defining the presence of EA [23, 24].

Ophthalmological and ENT procedures have been found to be independent risk factors for EA, especially in strabismus surgery and adenotonsillectomy surgery [7, 16, 17]. In literature, the incidence of EA among the pediatric population undergoing ENT surgery ranged between 8.2% and 50% [14, 25, 26], and ophthalmic surgery between 18.8% and 37% [27–30]. The overall incidence of EA in our study was 30%, with 34.5% for ENT and 24.4% for ophthalmic surgery. This incidence of EA in this study was compatible with results obtained from previous studies.

The EA in our study was more widely detected in patients in age group of 0–3 years (50%) and 4–6 years (30.4%) compared to patients more than 6 years (19.4%). These findings were consistent in other studies, which demonstrated that younger age is a risk factor for EA, particularly in preschool aged patients younger than 6 years old [17, 28]. Several studies have shown that male sex is associated with EA in adults, while the effect of sex on EA in children is not well known [17, 31]. Similarly, in our study, although male patients developed more EA than females, there was no significant difference.

With regard to anesthetic drugs, we did not compare volatile anesthetics with propofol as all patients received sevoflurane for anesthesia maintenance and almost all received both propofol and sevoflurane for induction. However, intraoperative opioids varied among participants. We found that participants who received hydromorphone had the lower incidence of EA (11.5%), compared with those who did not (36.5%). The difference was found to be significant on the univariate analysis but not on the multivariate analysis. Few studies reported the effect of hydromorphone on EA of children and the results were controversial [32, 33], consequently need further studies.

Binary logistics regression analysis found that high preoperative m-YPAS grade and high postoperative FLACC score were risk factors of EA which were consistent with previous study. Preoperative anxiety is generally accepted as a major risk factor for postoperative ED [31, 34]. Kain et al. reported a strong association between the incidence of EA and preoperative anxiety [35]. In addition, the higher pain score assessed with FLACC was found to increase the risk of EA. Pain is considered a major risk factor for EA in children [17], but it is difficult to distinguish between EA and behavioral manifestations due to postoperative pain [19, 36]. The descriptive behavior items of ‘crying’ and ‘inconsolability’ in the Watcha scale overlap the behavior that indicates acute pain in children in the early postoperative period [36]. One study has suggested that it may not be necessary to differentiate EA from pain, as the treatment with opioids is recommended as primary strategy for both of them [37]. However, another study has stated that management differs between the two. Pain is managed with an agent with analgesic properties whereas ED is primarily managed by an agent with sedative properties [36]. Noteworthily, in our study, very few children (6%) received rescue analgesic medication despite 24% of participants having severe pain with a FLACC score > 6. In our institution, the analgesia record was reviewed firstly when children presented with agitation. If the PACU bedside anesthesiologists considered that the dosage of analgesic medication used was sufficient, sedative drugs or non-pharmacologic interventions were alternatively administrated. The median FLACC scores in the ward were acceptable in both groups with only one child in each group manifesting pain. It would appear that pain was not detected and managed adequately which manifested as EA in current practice in PACU, and further the management of pain in the PACU should be the key to managing EA.

Our study did not find increased incidence of complications, prolonged duration of PACU stay or hospital stay in EA children, although this has been reported in other studies [8, 31, 38]. In addition, we found that EA patients need more pharmacological administration (i.e. propofol) and non-pharmacological intervention compared to non-EA patients, as these strategies have been demonstrated to be effective measures to treat EA [31].

### Limitation of this study

Since it was an observational study, it was difficult to make a controlled environment for patients. As a result, this study did not consider some essential factors, like volatile anesthetics, dexmedetomidine and analgesics. For example, our institution routinely used inhalation for anesthesia in children undergoing surgery, consequently, the findings could not extrapolate to children exposed to other anesthesia regimes. Also, FLACC was not used objectively to quantify and manage pain. Besides, we considered our study explorative, therefore, it should be somewhat more cautious on causality assumptions and absence of differences in complications.

## Conclusion

In summary, EA remains a significant problem during recovery from anesthesia that challenges the PACU care provider in terms of more pharmacologic and non-pharmacologic nursing care in children undergoing ophthalmic or ENT surgery. This study identified that preoperative anxiety and postoperative pain are risk factors of associated with EA. Preoperative anxiety assessment and management, and administration of adjunct analgesic treatments should be considered in the routine care.

## Data Availability

Data will be accessed upon request of the corresponding author.

## Abbreviations

ASA: American Society of Anesthesiologists
CI: Confidence interval
EA: Emergence agitation
ENT: Otorhinolaryngology
FLACC: Face,Legs,Activity,Cry,Consolability
IQR: Interquartile range
m-YPAS: Modified Yale Preoperative Anxiety scale
OR: Odds ratio
PACU: Post-anesthesia care unit
PAED: Pediatric Anesthesia Emergence Delirium
